# Synthesis and Evaluation of Plugging Gel Resistant to 140 °C for Lost Circulation Control: Effective Reduction in Leakage Rate in Drilling Process

**DOI:** 10.3390/polym16121658

**Published:** 2024-06-11

**Authors:** Peng Xu, Jun Yu, Lingzhi Xie

**Affiliations:** 1 Cooperative Innovation Center of Unconventional Oil and Gas, Yangtze University, Wuhan 430100, China; 15826692204@163.com; 2 Hubei Key Laboratory of Oil and Gas Drilling and Production Engineering, Yangtze University, Wuhan 430100, China; 3 National Engineering Research Center for Oil & Gas Drilling and Completion Technology, School of Petroleum Engineering, Yangtze University, Wuhan 430100, China; 4 Jingzhou Jiahua Technology Co., Ltd., Jingzhou 434023, China

**Keywords:** gel material, plugging agent, high temperature resistance

## Abstract

Gel plugging agents have become one of the preferred methods for plugging in complex and severe loss conditions during drilling due to their good adaptability to loss channels. To address the common issue of poor temperature resistance in gel-based plugging agents, high-temperature-resistant gel plugging materials were synthesized through the molecular design of polymers, modifying existing agents. Based on the temperature and salt resistance of the aqueous solution of an acrylamide (AM)/N-vinylpyrrolidone (NVP) binary copolymer, temperature-resistant monomer sodium styrene sulfonate (SSS) was introduced and reacted in a polyvinyl alcohol (PVA) aqueous solution. Using ammonium persulfate (APS) as an initiator and crosslinking with N,N-methylenebisacrylamide (MBA), a gel plugging material resistant to 140 °C was synthesized. The structure, thermal stability, water absorption and expansion, and plugging performance of the gel were studied through hot rolling aging, thermogravimetric analysis, infrared spectroscopy, electron microscopy scanning, sand bed experiments, and drag reduction experiments. The results show that the gel material has good thermal stability and water absorption and expansion at 140 °C, and its temperature-resistant plugging performance is excellent, significantly slowing down the loss rate of drilling fluid. This provides a basis for the further development of gel materials.

## 1. Introduction

The exploration and development of oil and gas fields have shifted to complex reservoirs such as shale oil and gas, tight oil and gas, and deepwater and deep oil and gas. The problem of drilling leakage has greatly reduced the drilling efficiency of complex wells. In particular, the malignant leakage in fractured formations has lost drilling time, consumed a large amount of plugging and drilling fluid materials, and greatly increased drilling costs, which has become a core technical problem in drilling engineering [1,2]. Currently, chemical plugging is the main method for drilling plugging, and physical plugging methods such as expansion tubes are being developed. High-temperature-resistant plugging materials are becoming a research hotspot, and gel materials for malignant leakage have also received great attention.

Drilling fluid plugging materials include high-filtration plugging materials, bridging plugging materials, polymer gel plugging materials, cement slurry plugging materials, and expansion plugging materials. As a highly utilized drilling fluid plugging material, high-filtration plugging materials are mainly composed of fibrous materials and diatomaceous earth. These plugging materials have a relatively strong toughness and plasticity, as well as strong compressive strength [3]. Bridging plugging materials are a type of plugging material that is frequently used and are composed of a combination of inert materials. These materials not only have the performance advantages of a single inert material, but also have low operational difficulty. The performance stability of these materials is high, and they are also compatible with the fluidity characteristics of drilling fluid [4]. Cement slurry plugging materials are a hybrid plugging material composed of lime, gypsum, and cement, which are mostly used in situations where there is severe leakage [5]. Expansion plugging materials are a mixture of crystalline polymers of different sizes and specific particulate materials, which undergo significant expansion after hydration. This expansion material can fully exert its plugging effect in a short period of time, usually for extremely severe leakage problems [6]. Polymer gel plugging materials have excellent advantages in plugging effectiveness. These plugging materials are composed of gel substances, which have relatively stable barrier properties. During the construction of well leakage plugging, the gel substances will penetrate all areas of the well leakage to control the expansion trend of fractures [7]. Under the influence of gel plugging, the plugging strength of the channel will be significantly enhanced, and most polymer gel plugging materials have excellent stability, which can help the material form a close fit with other materials due to their shear resistance and viscous resistance. As the material combines with other inert materials, its overall performance advantages will also increase significantly [8].

Due to its strong adaptability, gel is one of the commonly used plugging materials, which not only has a stable performance but also has an excellent plugging effect [9,10]. When the polymer gel material is combined with the leakage layer, its outer layer will gradually form a corresponding network structure. As this network structure spreads, its excess water will be absorbed, and the expansion of pores can be effectively controlled [11]. Under the dual effects of chemistry and physics, the internal structure of the polymer gel will also expand, and when it expands to the extent that it can plug the leakage layer, the expansion will stop [12,13,14]. Therefore, polymer gel plugging is also a relatively effective method. Many experts and scholars at home and abroad have conducted research on gel plugging materials. Vasquez et al. [15] synthesized a thermally stable gel at 120 °C using acrylamide and polyvinyl pyrrolidone; Salunkhe et al. [16] synthesized a robust ultra-high temperature hydrogel composition using monomers N,N-dimethylacrylamide and sodium styrene sulfonate; Hutchins et al. [17] formed a high-temperature-resistant and thermally stable gel system using polyacrylamide compatible with hydrogen sulfide-containing fluids; and Zhang et al. [18] developed a new type of high-temperature-resistant fiber-reinforced gel plugging agent using acrylamide, butyl methacrylate, and 2-acrylamido-2-methylpropane sulfonic acid as monomers and flexible fibers as reinforcement materials.

For high-temperature formations, high temperatures tend to lose their effect, leading to the failure of the gel material, making it difficult to effectively seal the leakage channels in the formation [19,20,21]. Considering the temperature resistance of monomers and the molecular design of polymers, on the basis that conventional acrylamide (AM) can provide C-C as the main chain, N-vinylpyrrolidone (NVP), a temperature-resistant monomer with a non-ionic group and strong hydrolysis resistance, sodium styrenesulfonate (SSS), which can provide a stable adsorption group at a high temperature—sulfonic group—and polyvinyl alcohol (PVA), which can provide an aqueous solution environment with temperature-resistance coating, are introduced to crosslink with an N,N-methylenebisacrylamide (MBA) crosslinker, using ammonium persulfate (APS) as the initiator, to synthesize a temperature-resistant gel plugging material that can provide some reference for the study of malignant leakage in fractured formations.

## 2. Materials and Methods

### 2.1. Materials

(1) Experimental reagents: acrylamide (AM), analytical pure; N-vinyl pyrrolidone (NVP), containing 100 ppm NaOH stabilizer; N, N-methylene bisacrylamide (MBA), all purchased from Shanghai Aladdin Biochemical Technology Co., Ltd. (China); sodium styrene sulfonate (SSS), purchased from Shanghai Bohr Reagent Co., Ltd. (China); polyvinyl alcohol (PVA), purchased from Shanghai Chenqi Chemical Technology Co., Ltd. (China); ammonium persulfate (APS), analytical pure, purchased from Tianjin Damao Chemical Reagent Factory (China); distilled water, self-made in the laboratory.
(2) Experimental instruments: vacuum drying oven, Shanghai Shangpu Instrument Equipment Co., Ltd. (China); magnetic stirrer and rotary stirrer, Jiangsu Jincheng Guosheng Experimental Instrument Factory (China); roller heating furnace, OFI Testing Equipment, (Houston, TX, USA); electric thermostatic water bath, Shanghai Yulong Instrument Equipment Co., Ltd. (China); pressure reducing valve, Qingdao Huaqing Automation Instrument Co., Ltd. (China); pressure gauge, Xi’an Automation Instrument Factory (China); three-type piston container and confining pressure pump, Nantong Yichuang Experimental Instrument Co., Ltd. (China); electronic balance, Harbin Zhonghui Weighing Apparatus Co., Ltd. (China); multi-function grinder, Beijing Yongguangming Medical Instrument Co., Ltd. (China); thermogravimetric analyzer, Nanjing Huicheng Instrument Co., Ltd. (China); infrared spectrometer, Brook Corporation, (Boston, MA, USA); cold field emission scanning electron microscope, HITACHI, Tokyo (Japan); MD-NJ5 (5961) gel strength tester, Zhejiang Fengyuan Electronics Co., Ltd. (China); visual sand bed sealing device and high temperature high pressure filtration instrument, Qingdao Chuangmeng Instrument Co., Ltd. (China).

### 2.2. Experimental Methods

#### 2.2.1. Synthesis Method of Gel

The drug dosage is increased according to the mass ratio, and the gel is synthesized by aqueous solution polymerization.

(1) Weigh 7 g of 1788 mesh PVA and add it to 100 g of distilled water. Stir thoroughly at room temperature using a magnetic stirrer until fully dissolved, then set aside for later use;
(2) Weigh 30 g of AM and dissolve it in 100 g of distilled water. Then, add 20 g of NVP and 10 g of SSS, stir well and dissolve;
(3) Transfer the dissolved PVA solution and AM, NVP, SSS solution into a three-neck flask and mix them thoroughly. Add 0.5 g APS and 0.65 g MBA and react at 60 °C for 5 h under a nitrogen-protected environment. Maintain a rotation speed of 600 r/min during the experiment.

After the reaction is fully completed, the product is cooled to room temperature and taken out and dried in an oven at 70 °C. Then, it is crushed and sieved using a universal crusher to obtain uniform particle size gel dry powder particles.

**Figure 1:**
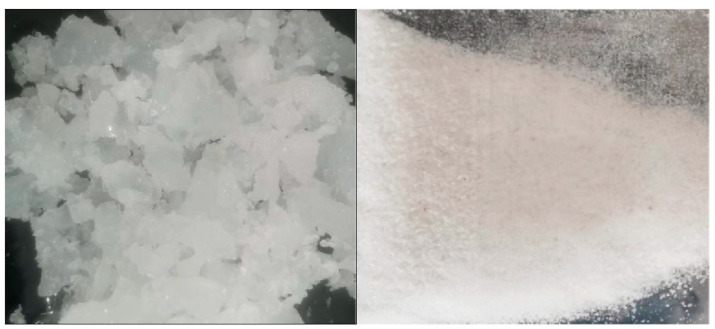
State of gel products before drying and crushed state of gel products after drying.

The state of gel products before drying and crushed state of gel products after drying are shown in Figure 1.

#### 2.2.2. Characterization of Gel

(1) Infrared characterization of gel (FT-IR)

In order to determine the target product of the gel particles, it was characterized by infrared, according to the national standard GB/T 6040-2002 [22]. General rules for infrared spectral analysis methods—Part 5: Sample preparation method, the ground gel dry powder particles were mixed with potassium chloride to prepare samples. The infrared spectral characteristics of the polymer were determined by infrared spectrometer, and the wave number range was 400~4000 cm^−1^.

(2) Thermogravimetric analysis of gel

The mass change in the composite gel was determined using a thermogravimetric analyzer in a nitrogen environment at a heating rate of 10 °C/min, with a testing temperature range of (25~800 °C), protected by inert gas, and a heating rate of 10 °C/min, and the thermal stability study was carried out using a differential thermal thermogravimetric synchronous analyzer, according to the petrochemical industry standard of the People’s Republic of China, NB/SH/T 0859-2013 [23]. Thermal analysis method was used for the determination of thermal stability of chemical substances, and the TG curves and corresponding data obtained from the experiments were analyzed to evaluate the temperature resistance.

(3) Gel scanning electron microscope analysis

The hydrogel samples were placed in a freeze-drying machine and freeze-dried under vacuum conditions. Then, the freeze-dried sample was processed and crushed into powder, and then the quantitative microsphere powder was scanned by electron microscope, according to GB/T 36422-2018 [24]. Determination of micromorphology and diameter by scanning electron microscope and the microscopic analysis of electron microscope was carried out.

#### 2.2.3. Evaluation of Water-Swelling Performance of Gel

(1) Evaluation of water absorption of gel

Weigh 1 g of gel dry powder particles with an average particle size of 850 μm, place them in an aging tank, add 500 mL of water, and place them in a roller heating furnace. After fully aging for the designed time at a high temperature of 140 °C, weigh the filter screen. The ratio of the difference in mass before and after aging to the mass of the gel particles before aging can be used to obtain the expansion coefficient at high temperatures.

(2) Gel strength analysis after aging

Place the gel after aging at 140 °C for different periods of time in a measuring container of a gel strength tester, design a fixed volume and a fixed indentation depth, and then measure the gel strength values corresponding to different aging times.

#### 2.2.4. Evaluation of the Filtration Property of Gel

(1) Temperature range test of the gel

Place 10 g of gel dry powder particles with an average particle size of 850 μm in an aging tank, add 500 mL of water, and place it in a roller heating furnace. Set the temperature to 120 °C, 130 °C, 140 °C, and 150 °C for 24 h of aging. Remove and observe the changes in morphology and quality, as well as the loss of the gel after aging. The gel still has a good morphology at 140 °C, but begins to dissolve and lose mass at 150 °C.

(2) Evaluation of gel blocking performance

Pour the quartz sand with a particle size of 20–40 mesh into the plexiglass tube (inner diameter 5 cm) of the visualized sand bed blocking device, so that the quartz sand is filled to the glass tube at the 350 mL scale and compacted. The gel was hot rolled at 140 °C for 18 h, 24 h, and 30 h to plug the sand bed, and the plugging performance of the gel was evaluated by recording the filtration rate of the drilling fluid and comparing the change in the base slurry.

(3) Gel drag reduction evaluation

A PU air pump pressure-bearing tube with an inner diameter of 6.5 mm and an outer diameter of 10 mm was selected, and the pressure-bearing tube was 2 m long. The time was measured for the clear water string, drilling fluid string, guar gum string, and aged gel string (the fixed length of the string was 15 cm) through a 2 m long pressure-bearing tube under a fixed pressure of 0.1 MPa.

(4) High-temperature and high-pressure filtration performance evaluation of gel

Use a high-temperature and high-pressure filtration tester to measure the change in high-temperature and high-pressure filtration loss of the drilling fluid before and after adding gel particles at a temperature of 120 °C and a pressure of 3.5 MPa to evaluate the high-temperature and high-pressure filtration performance of the gel.

#### 2.2.5. Polymerization Mechanism of Gel

For high-temperature-resistant treatment agents, it is necessary to have strong thermal stability, no obvious degradation at the temperature used, and easy control of crosslinking. This requires that in the molecular structure, the main chain or hydrophilic group and the main chain connecting bond as far as possible to use C-C, its bond energy is high, has good stability at high temperatures, and is not prone to fracture due to high-temperature effects; in order to improve the stability of functional groups on the side chain in a high-temperature environment, the structure of a C-N or C-S bond with high bond energy and good thermal stability is selected as the side chain [25]. N,N-methylenebisacrylamide (MBA) is a commonly used monomer polymerization organic crosslinking agent, its active crosslinking group is a C-C bond, which has the characteristics of multi-point initiation, and the organic crosslinking agent has large bond energy and good stability [26]. Acrylamide (AM) is a monomer commonly used in the synthesis of high-temperature-resistant plugging materials, which is chemically stable and can provide a thermally stable C-C linkage to the main chain [27]. N-vinylpyrrolidone (NVP) provides thermally stable C-C linkages and C-N bonds to the main chain; sodium p-styrene sulfonate (SSS) also provides C-C linkages and contains -SO_3_^−^, which is thermally stable, hydrophilic, and able to function at lower pH values. Polyvinyl alcohol (PVA) does not melt when heated and undergoes water loss decomposition at 150 °C. Its aqueous solution has good adhesive and film-forming properties, which can effectively slow down thermal decomposition, and also provide thermally stable C-C connecting bonds.

**Figure 2:**
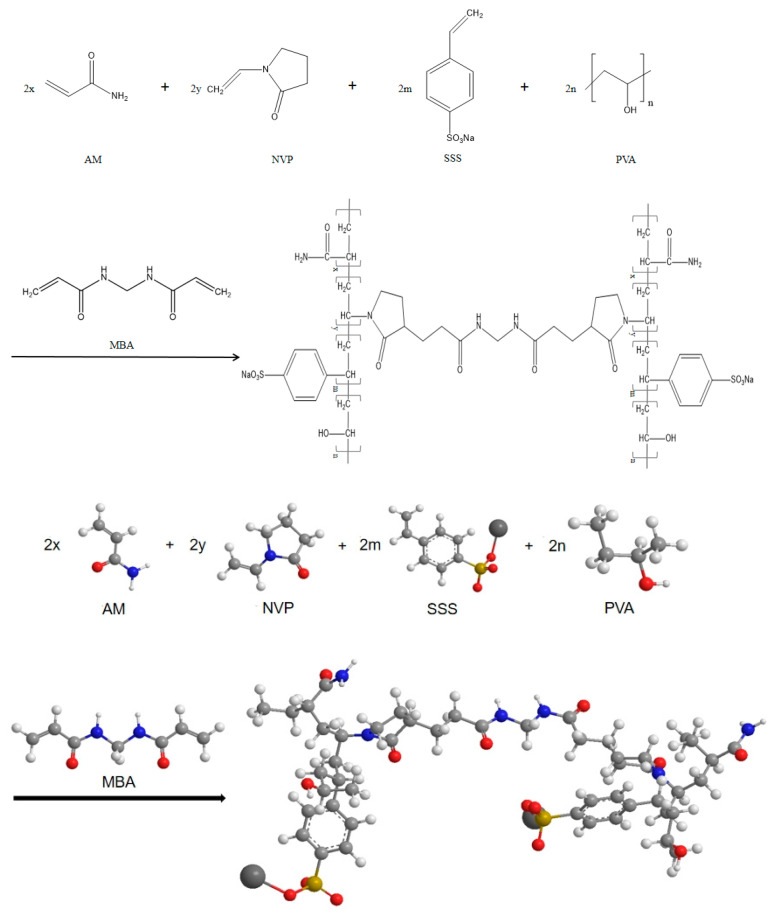
Schematic diagram of the polymerization reaction of AM-NVP-SSS-PVA copolymer.

The performance of the polymer can be effectively improved by introducing a monomer with special functionality to copolymerize with acrylamide (AM). Acrylamide (AM) can provide a non-ionic group such as an amide group, which has strong adsorption capacity and can improve the overall performance of the polymer. N-vinylpyrrolidone (NVP) has good hydrolysis resistance at high temperatures, which can provide stable adsorption groups at high temperatures and can be used as a non-ionic monomer that can provide hydration groups, and has good hydrolysis stability at high temperatures. And it can inhibit the hydrolysis of the amide group on the molecular chain; in the case of higher temperature requirements of the treatment agent, the proper introduction of this monomer can be very good to increase the temperature resistance of the polymer [28,29,30,31]. Sodium p-styrene sulfonate (SSS) is a hydrophilic anionic monomer, which can fully participate in the polymerization reaction in aqueous solution and has sulfonic acid groups. The presence of two S-O (Π bond) bonds of the sulfonic acid group enhances the ability of S to attract electrons from -OH, making it easier for S to attract electrons from -OH to produce the conjugation effect of the -SO_3_^−^ system, making the sulfonic acid group more stable. At the same time, its unique benzene ring structure can improve the rigidity of the molecular chain, and the appropriate introduction can help to improve the temperature resistance of the polymer [32]. Polyvinyl alcohol (PVA) can provide hydroxyl groups, which are resistant to hydrolysis, have strong adsorption capacity, and have strong temperature resistance [33]. The molecular structure diagram of the synthesized copolymer is shown in Figure 2.

#### 2.2.6. Plugging Mechanism of Gel

**Figure 3:**
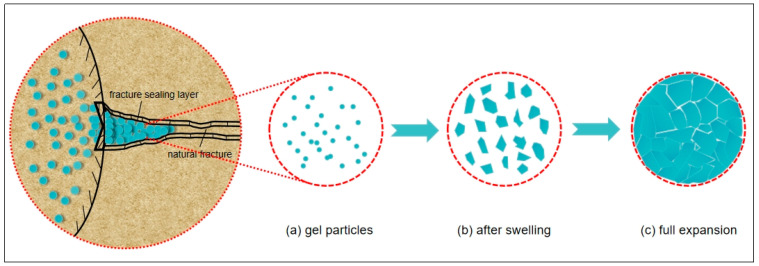
Schematic diagram of the principle of plugging with gel particles.

The gel particles enter the fracture, absorb water and expand in the fracture, squeeze and fill, and the plugging mechanism is shown in Figure 3. Due to the pressure difference, the gel particles enter the fracture with the fluid and the fracture channel is characterized by roughness, non-homogeneity, and different sizes. The gel enters the leakage channel in the form of small particles, which are not affected by the shape and size of the channel. The gel particles are retained in contact with the fracture wall, and the gel particles are continuously squeezed and accumulated. After water absorption and expansion, the volume of the gel particles continues to increase. The particles squeeze and fill each other until they fill the whole fracture channel, producing an expansion and filling effect on the leakage layer fractures, forming a local relatively stable blocking layer. Due to the ductility of the gel, with the increase in pressure, the gap between the gel particles is further reduced, and eventually compacted to form a dense pressure-bearing plugging layer [34,35].

The surface area of the gel increases after absorbing water and swelling, the contact area with the fracture wall becomes larger, and the retention capacity becomes stronger, while the gel has a mutual squeezing force and viscoelasticity between the gel and the gel in the fracture channel, and it can produce a large reaction force on the inner wall of the fracture [36]. At the same time, in the high-temperature environment, the gel still has a certain strength and good pressure-bearing capacity.

## 3. Results and Discussion

### 3.1. Characteristics of Gel

#### 3.1.1. FT-IR

**Figure 4:**
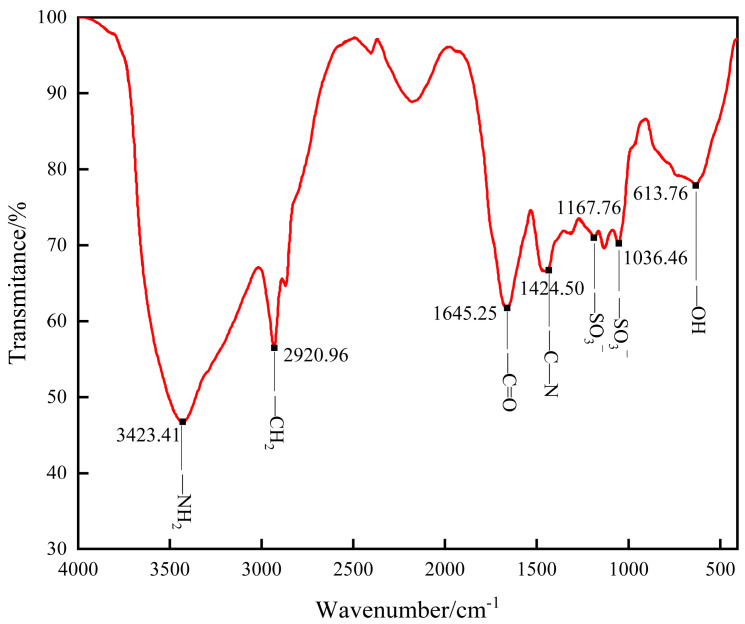
Infrared spectra of the gel.

The infrared spectrum of the gel is shown in Figure 4. It can be seen that the stretching vibration absorption peak of -NH_2_ is at 3423.41 cm^−1^, and the stretching vibration absorption peak of -C=O is at 1645.25 cm^−1^. These two peaks indicate that the polymer monomer AM has successfully participated in the reaction. At 1424.50 cm^−1^, there is a stretching vibration absorption peak of -C-N, indicating that NVP has successfully participated in the reaction. The bending vibration absorption peak of -OH is at 613.76 cm^−1^, and the stretching vibration absorption peak of -CH_2_- is at 2920.96 cm^−1^. These two peaks indicate that PVA has successfully participated in the reaction. The bending vibration absorption peaks of -SO_3_^−^ were observed at 1167.76 cm^−1^ and 1036.46 cm^−1^, indicating that SSS successfully participated in the reaction. The analysis shows that AM, NVP, PVA, and SSS have successfully polymerized and crosslinked, and the synthesized gel plugging agent is the target product.

#### 3.1.2. TGA

**Figure 5:**
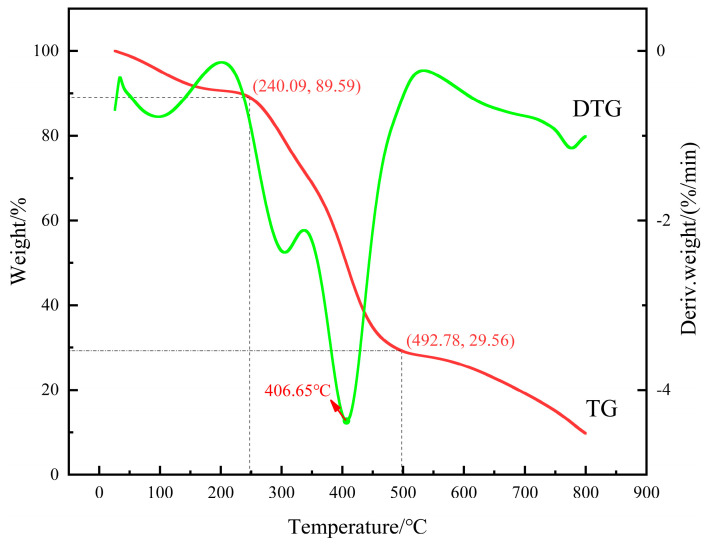
Thermogravimetric analysis curve of the gel.

The thermal gravimetric analysis curve of the gel is shown in Figure 5. From the curve TG, it can be seen that the weight loss of the gel is divided into three stages: 0~240.09 °C, 240.09~492.78 °C, and 492.78~800 °C. In the first stage, from 0 °C to 240.09 °C, as indicated by the point (240.09, 89.59) on the figure, it can be observed that the remaining weight percentage at a temperature of 240.09 °C is 89.59%, with a weight loss of 10.41%. The weight loss in this stage is mainly caused by the evaporation of residual moisture in the gel, accounting for about 10.41% of the total weight loss. In the second stage, from 240.09 °C to 492.78 °C, as indicated by the points (240.09, 89.59) and (492.78, 29.56), it can be seen that the weight loss of the gel is more significant in this temperature range, accounting for about 60.03% of the total weight loss. This is mainly due to the longer main chain and side chains of the synthesized gel, which are easily decomposed and destroyed in a high-temperature environment, resulting in the loss of side-chain weight. The third stage is from 492.78 °C to 800 °C. From the point (492.78, 29.56), it can be seen that after the temperature rises to 492.78 °C, the main chain begins to break, and the crosslinking points between molecules break, gradually losing thermal resistance. From the figure, it can be seen that the gel has good temperature resistance and thermal stability at temperatures not exceeding 240.09 °C.

#### 3.1.3. Gel Scanning Electron Microscopy Analysis

**Figure 6:**
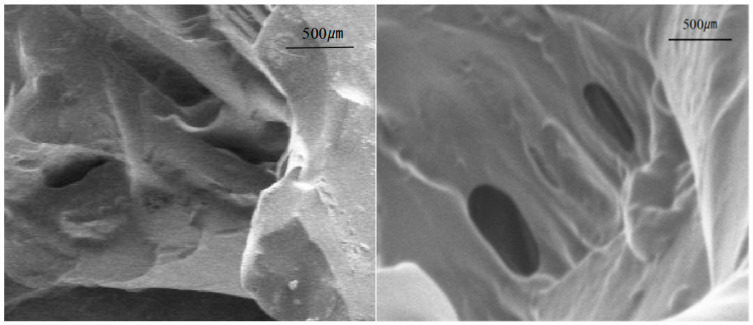
Scanning electron microscope of the gel.

The state of the gel presented in the microstructure greatly affects the efficiency of gel plugging and delay. Figure 6 shows the electron microscope scanning image of the gel after absorbing water and swelling and freeze-drying at a high temperature of 140 °C. It can be seen from the figure that the synthesized gel shows a good three-dimensional space network structure. The gel polymerization network is relatively relaxed, and the surface area of the gel is large with a certain number of connected pores distributed on the surface. The properties of the synthesized gel make it have a better expansion rate after entering the fracture leakage channel, faster water absorption rate, larger water absorption expansion coefficient, and good swelling and ductility. The material has projections and a large surface area, indicating strong interaction forces between the polymer molecules and excellent temperature resistance and viscoelasticity.

### 3.2. Analysis of Water-Swelling Performance of Gel

#### 3.2.1. Water Absorption and Expansion of Gel

**Figure 7:**
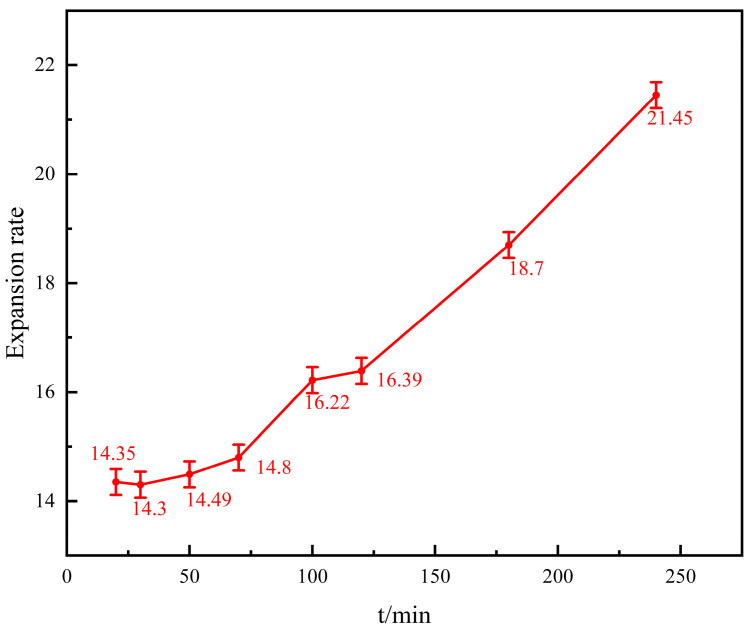
Water-swelling rate curve of the gel.

Take 1 g of gel dry powder particles with an average particle size of 850 μm after crushing, divided into eight groups, and aged by a roller heating furnace at 140 °C for 20 min, 30 min, 50 min, 70 min, 100 min, 120 min, 180 min, and 240 min, respectively, and then filtered dry after measuring the mass after water absorption, and comparing it with the mass before water absorption, to calculate the expansion property of its water absorption. The difference between the mass before and after and the mass of the gel particles before aging can be compared to the expansion coefficient at high temperatures. The relationship between the mass of the gel particles before and after aging and water absorption at different times is shown in Figure 7.

It can be seen from Figure 7 that the expansion ratio of the gel increases from 14.35 ratios at the beginning to 21.45 ratios as the aging time increases under a high-temperature environment. As the temperature rises, the molecular thermal movement intensifies, the gel network structure gradually expands at high temperatures, and the water molecules enter the grid structure, which is shown by the increase in the water absorption ratio of the gel. With the increase in the aging time, the gel water absorption gradually reached saturation, and the molecular thermal movement also tended to balance. The size of the gel particles increased significantly after high-temperature water absorption, showing a good high-temperature water absorption performance.

**Figure 8:**
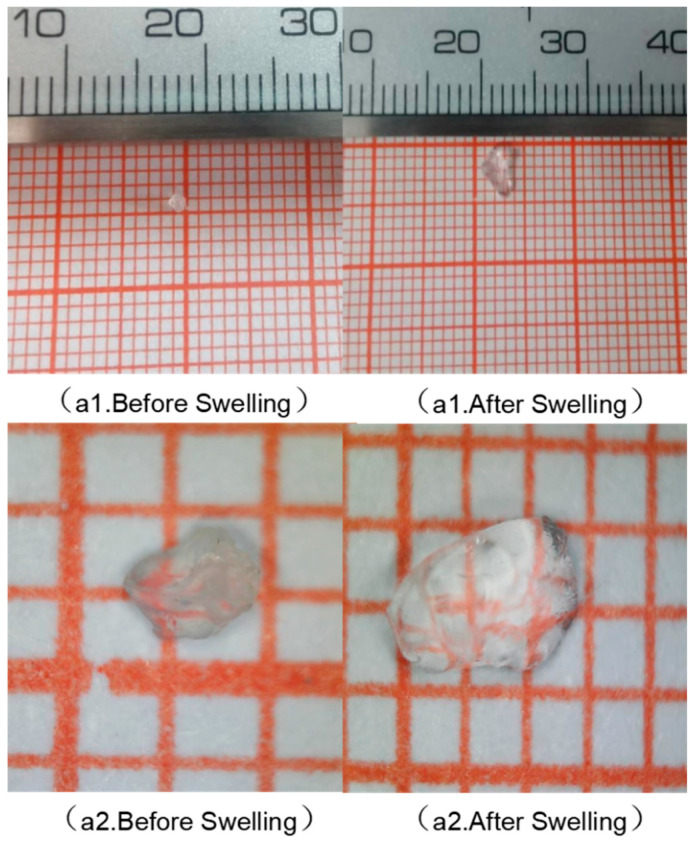
Comparison chart of gel before and after water absorption. (**a1**) is a comparison of the morphology observed by the naked eye; (**a2**) is a comparative image of the morphology under an electron microscope.

With the help of the Supereyes electron microscope and the comparison of vernier caliper, the comparison diagram of the gel particles before and after water absorption obtained under the naked eye and electron microscope is shown in Figure 8. It can be seen from the figure that the gel particles before water absorption occupy more than 1.5 grids, and the volume of the gel particles after full water absorption is about 6 grids. The macro performance is that the volume of gel after water absorption increases, and the volume after water absorption can reach four ratios more than before water absorption, fully showing the water absorption expansion performance of the gel. The gel particles have the ability of water absorption and expansion, which can meet the purpose of forming a sealing layer after entering the leakage channel and filling the entire channel with volume expansion.

#### 3.2.2. Strength Analysis of Gels after Water Absorption

**Figure 9:**
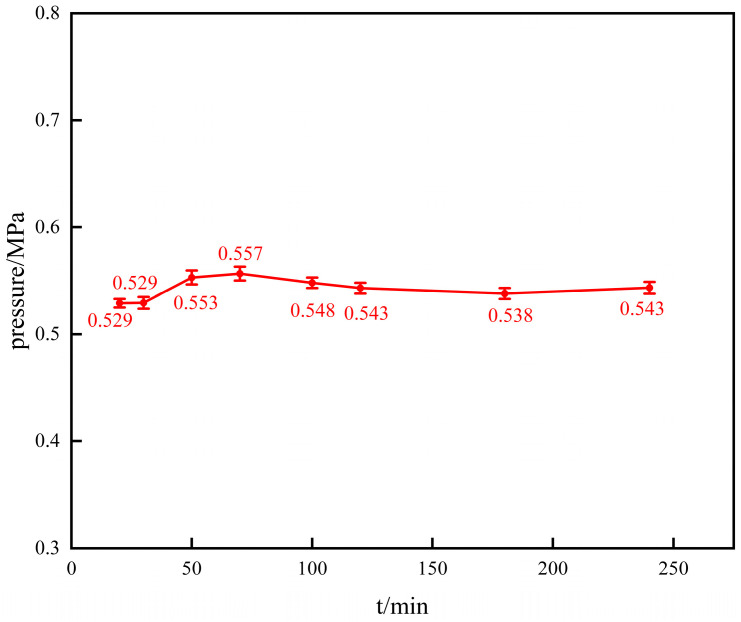
Changes in gel strength after different aging times.

Take 1 g of gel dry powder particles with an average particle size of 850 μm after crushing, and age them for 20 min, 30 min, 50 min, 70 min, 100 min, 120 min, 180 min, and 240 min in a roller heating furnace at 140 °C. After filtering through a filter screen, place them in a gel strength tester. The equipment model of the gel strength tester is MD-NJ5, with a maximum load of 50 N, a test speed of 2 cm/min adjustable speed, a test stroke of 0–200 mm, and an equipment number of 5961. The gel strength results after different aging times are shown in Figure 9.

As shown in Figure 9, the gel’s pressure-bearing capacity after aging for 20 min, 30 min, 50 min, 70 min, 100 min, 120 min, 180 min, and 240 min is 0.529 MPa, 0.529 MPa, 0.553 MPa, 0.557 MPa, 0.548 MPa, 0.543 MPa, 0.538 MPa, and 0.543 MPa, respectively. The pressure-bearing strength values of the gel do not change significantly and are relatively stable. The strength of the gel after fully absorbing water is basically stable within the range of 0.5–0.6 MPa, indicating that the gel particles have a relatively stable pressure-bearing capacity.

### 3.3. Filtration Property of Gel

#### 3.3.1. Effect of the Temperature

**Figure 10:**
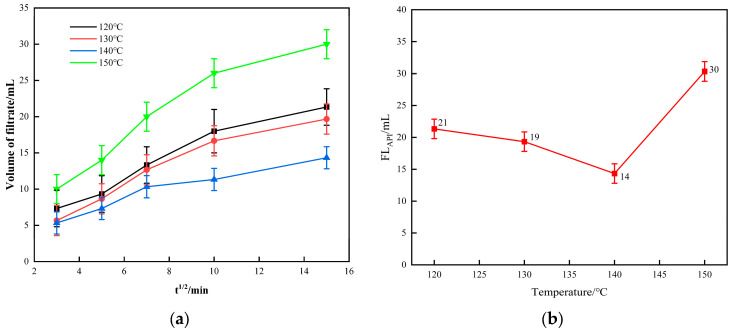
(**a**) The filtration rate; (**b**) FLAPI of bentonite containing 4.0% gel particles at different temperatures.

When the gel particle concentration was fixed at 4.0%, the effect of the temperature variation on the fluid loss performance from 120 °C to 150 °C was evaluated, as shown in Figure 10.

The analysis shows that the gel particles were tested at 120 °C, 130 °C, 140 °C, and 150 °C. From Figure 10b, it can be seen that the fluid loss of the bentonite slurry containing gel particles was 14 mL at 140 °C, which exhibited the best fluid loss reduction performance compared to the fluid loss at 120 °C, 130 °C, and 150 °C. At 150 °C, the gel particles began to lose their ability to reduce fluid loss, with the FL_API_ and filtration rate starting to increase. Additionally, at 140 °C, the bentonite had the lowest filtration rate and fluid loss, indicating that the gel particles had the best filtration reduction performance. This is possibly because the gel has suitable softening conditions at 140 °C, showing excellent plugging effects. When the temperature rises to 150 °C or higher, the gel begins to melt and decompose, leading to poor filtration performance.

#### 3.3.2. Evaluation of Sand Bed Plugging Performance

**Figure 11:**
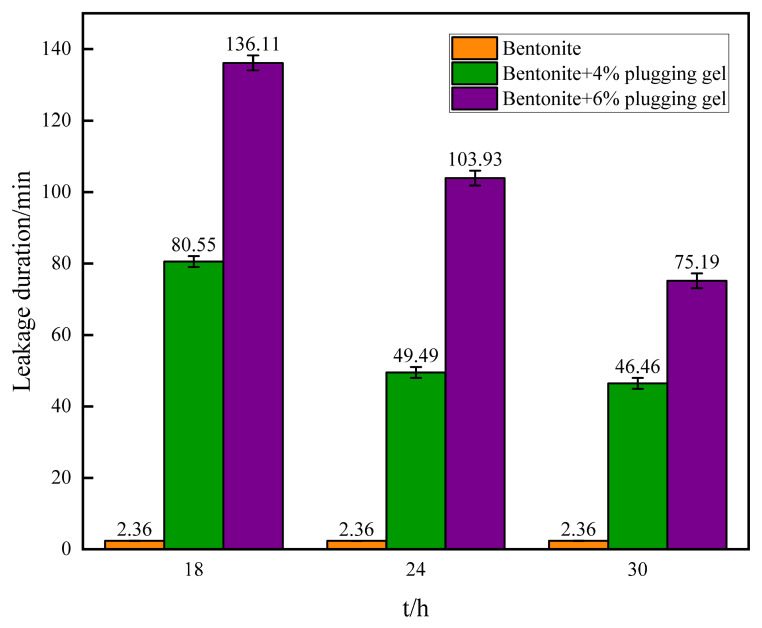
Comparison of sand bed plugging loss by the gel.

After aging the gel at 140 °C for 18 h, 24 h, and 30 h, sand bed plugging experiments were conducted, as shown in Figure 11. The results indicate that compared to the base slurry, the fluid loss rate significantly decreased after adding gel particles. The higher the gel content, the better the plugging effect. This is because the gel, after aging and absorbing water, becomes flexible and fills the pores of the sand bed along with other solid phases in the drilling fluid under pressure, thus slowing down the fluid loss. The figures show that even after continuous aging at 140 °C for 18 h, 24 h, and 30 h, the gel particles still exhibit good fluid loss reduction effects and have a long-lasting temperature-resistance performance.

#### 3.3.3. Evaluation of Drag Reduction in Simulated Fractured Pipelines

**Figure 12:**
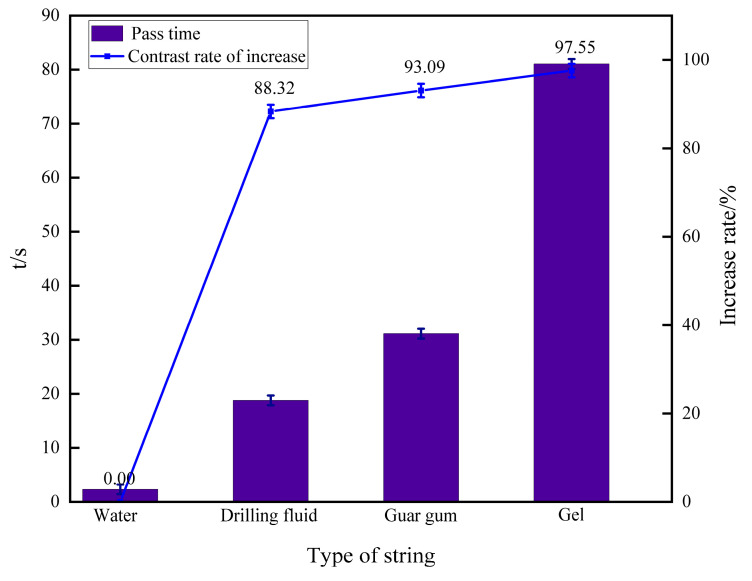
Time of passage of different fluids through the pipe.

Maintain the pressure at a fixed 0.1 MPa using a pressure control valve. The time for various fluid columns to pass through the pressure-bearing pipeline is shown in Figure 12. Due to the smooth pipeline, the water column passed through quickly under pressure, taking 2.32 s; the drilling fluid column took 18.8 s; the 1% concentration guar gum column took 31.14 s; and the gel column took 81.07 s. As shown in the figure, the gel column has a significant drag reduction effect compared to the other three columns, improving by 97.55% compared to water. This indicates that after entering the rough and heterogeneous fracture channels, the gel can have sufficient time to contact and adhere to the fracture walls, gradually forming a plugging layer, thus slowing down the fluid loss.

#### 3.3.4. High-Temperature and High-Pressure Filtration Loss Evaluation

**Figure 13:**
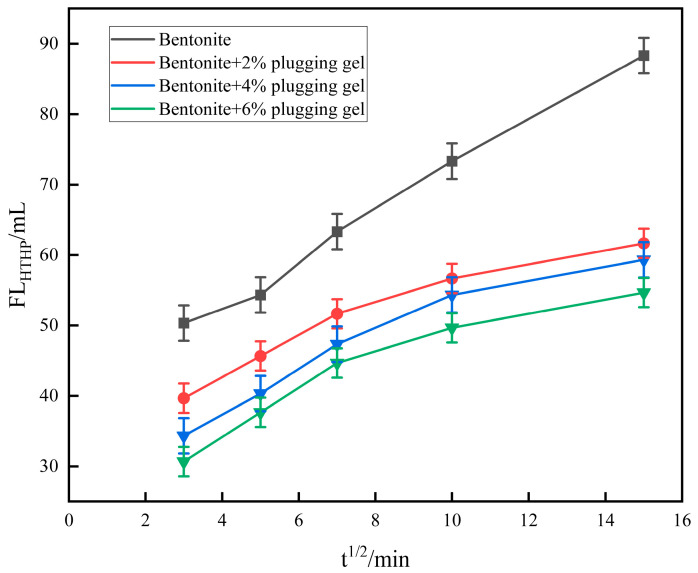
Changes in high-temperature and high-pressure filtration loss of gels at different concentrations.

The temperature is controlled at 140 °C, and the differential pressure between the top and bottom of the drilling fluid cup is 3.5 MPa. The changes in HTHP filtration before and after adding different concentrations of gel into the drilling fluid are shown in Figure 13.

It can be seen from Figure 13 that the filtration loss is effectively reduced after adding gel, and the volume of gel with different concentrations to reduce the filtration loss is different, which shows that the higher the concentration of gel that is added, the more obvious the filtration loss reduction effect is, indicating that gel can still play a role in delaying the leakage under a high temperature and pressure.

## 4. Conclusions

(1) An anti-140 °C high-temperature gel plugging material was synthesized by aqueous solution polymerization with acrylamide, N-vinylpyrrolidone, sodium p-styrene sulfonate, and polyvinyl alcohol as polymerization monomers, N,N-methylenebisacrylamide as the crosslinking agent, and ammonium persulfate as the initiator.
(2) The gel material has a good high-temperature water absorption and expansion. The size of the gel particles increases significantly after high-temperature water absorption. The expansion rate after water absorption can reach dozens of times more than before water absorption, and the gel strength performance is relatively stable. After 1 h, 2 h, and 4 h of high-temperature water absorption, the gel strength changes little and is stable between 0.4 and 0.6 MPa.
(3) The gel material has a good temperature resistance and plugging performance. After aging at 140 °C for 24 h, it basically does not degrade. It has long-term temperature resistance and can maintain effective plugging for a long time at high temperatures. After 18 h, 24 h, and 30 h of aging, the plugging effect is significantly improved compared with the base slurry.
(4) After aging, the gel material has good drag reduction, which is 97.55% higher than that of clear water. It can fully contact and retain with the fracture wall, squeeze and accumulate, and finally form a plugging layer to achieve the purpose of plugging. The gel material also has a significant filtration reduction effect on drilling fluid under high-temperature and high-pressure conditions.

## Data Availability

The data are contained within the article.
